# Diet, physical activity, and emotional health: what works, what doesn’t, and why we need integrated solutions for total worker health

**DOI:** 10.1186/s12889-020-8288-6

**Published:** 2020-01-31

**Authors:** Iffath U. B. Syed

**Affiliations:** School of Health Policy and Management, York University, Stong College, 3rd Floor, 4700 Keele Street, Toronto, Ontario M3J 1P3 Canada

**Keywords:** Immigrant health, Visible minorities, Public health, Social determinants of health, Total worker health

## Abstract

**Background:**

Current research advocates lifestyle factors to manage workers’ health issues, such as obesity, metabolic syndrome, and type II diabetes mellitus, among other things (World Health Organization (WHO) Obesity: preventing and managing the global epidemic, 2000; World Health Organization (WHO) Obesity and overweight, 2016), though little is known about employees’ lifestyle factors in high-stress, high turnover environments, such as in the long term care (LTC) sector.

**Methods:**

Drawing on qualitative single-case study in Ontario, Canada, this paper investigates an under-researched area consisting of the health practices of health care workers from high-stress, high turnover environments. In particular, it identifies LTC worker’s mechanisms for maintaining physical, emotional, and social wellbeing.

**Results:**

The findings suggest that while particular mechanisms were prevalent, such as through diet and exercise, they were often conducted in group settings or tied to emotional health, suggesting important social and mental health contexts to these behaviors. Furthermore, there were financial barriers that prevented workers from participating in these activities and achieving health benefits, suggesting that structurally, social determinants of health (SDoH), such as income and income distribution, are contextually important.

**Conclusions:**

Accordingly, given that workplace health promotion and protection must be addressed at the individual, organizational, and structural levels, this study advocates integrated, total worker health (TWH) initiatives that consider social determinants of health approaches, recognizing the wider socio-economic impacts of workers’ health and wellbeing.

## What is already known about this subject?

Current research advocates lifestyle factors to manage health issues of workers, such as managing obesity, metabolic syndrome, and type II diabetes mellitus, among other things [50, 51], though little is known about employees’ lifestyle factors in high-stress, high turnover environments, such as in the long term care (LTC) sector.

## What are the new findings?

The findings suggest that LTC workers typically maintained health and wellbeing through self-care measures conducted in group settings or tied to proxies of relaxation and emotional health, however, a few indicated that finances were barriers to practicing healthy behaviors.

## How might this study impact policy or practice in the foreseeable future?

This study impacts policy such that it advocates integrated, total worker health (TWH) initiatives that consider social determinants of health approaches, recognizing the wider socio-economic impacts of workers’ physical, mental, and social wellbeing.

## Background

A broad set of public health interventions have been advocated in the occupational health literature. These are generally accepted principles and practices that often include interventions that focus on prevention of accidents and occupational diseases, and also health promotion activities such as enabling workers to become more skilled to improve their lifestyles, quality of diets, sleep, and physical fitness [23]. For example, there is evidence that workers who experience high stress and job strain, as measured by the Siegrist effort rendered imbalance (ERI) model, have higher odds of having metabolic syndrome [25]. Furthermore, occupational stress, as measured by the psychological injury risk indicator (PIRI), is associated with metabolic syndrome components such as hypertriglyceridemia and high blood pressure [22]. Sleep hygiene is also important for workers’ health, safety, well-being, and productivity [24]. It is beneficial for emotional health, and it may reduce cardiovascular risk from metabolic syndrome [24]. Indeed, sleep problems are associated with: an increase in the incident cases of metabolic syndrome in high-stress occupational groups, such as police officers, in a prospective 5-year study [17], as well as workplace violence [24].

Other researchers have specifically advocated for the following: smoking cessation, consuming limited or no amount of alcohol, medical surveillance and screening of high risk and vulnerable groups; reducing chemical, physical, ergonomic and emotional exposures; increasing research and development of appropriate drugs and therapeutic products; and advocating interventions that include urban environmental interventions like building safe walkways, bicycle paths, and improving building design to encourage stairwell use [18, 35, 36]. The rationale for implementing workplace health promotion and protection interventions is because they provide intangible benefits such as increased job satisfaction and worker wellbeing [23].

As indicated by Magnavita [23]; workplace health promotion programs are designed so that they can help workers become more skilled in managing their chronic conditions; and proactive in their health-care by improving their lifestyles, quality of diet, and physical activity, among other things. Given that there is an increasing body of evidence which supports that employment and working conditions contribute to health problems previously considered unrelated to work, such as obesity [21, 30, 46], metabolic syndrome [22, 25], cardiovascular disease [20], sleep disorders [5, 17, 24], and depression [40], this study aimed to explore a new and under-researched area of health behaviors in high-stress, high-turnover work environments, such as in the long term care (LTC) sector.

Previous studies of Canadian LTC have focused on: structural and physical violence [3, 9]; work-related injuries and illness [1]; and presence of other health-limiting circumstances such as job stress, high workloads, labor intensification, task orientation, assembly-line style of work, work hierarchies, and strict divisions of labor [2, 10, 47]. However, no studies to date have examined the reporting of diet and physical activity among Canadian LTC workers. For this study, the research questions asked: To what extent do LTC workers report diet and physical activity as mechanisms to maintain their health and wellbeing? How do LTC workers maintain emotional health? The rationale for this study’s focus on diet and physical activity is to establish a starting point given that such health protection and promotion efforts are under-researched areas in Canadian LTC.

## Methods

This study used a single-case design, and relied on an ethnography, in which the sources of evidence were from direct observations and in-depth, key informant interviews. Observations and interviews were carried out at a LTC site in Toronto, Canada between the hours of 6:30 am am to midnight, which were opportunistic times to observe the workers’ scheduled shift changes that occurred at 7 am, 3 pm, and 11 pm. Observations were conducted in secure, locked and unlocked units/ wings at the site; in public spaces within the facility, and at the reception area. These spaces included: hallways and dining areas on the individual units, the recreation space of the atrium located on the ground floor, the employee break room located at the mezzanine level, and outside the meeting rooms in the basement-level. Forty-two face-to-face, in-depth, semi-structured interviews were conducted with participants, and digitally recorded using Sony ICDPX440 recorders. Fieldnotes were generated during observations, which documented preliminary thoughts, assumptions, and the physical setting. Multiple units of analysis were organized by worker characteristics such as sex, job titles or roles, visible minority (VM) status, full time (F/T) status, and part-time (P/T) work status, among other things (Table 1). Fieldnotes and interview transcripts were analyzed with thematic analysis for the study using a coding system with the aid of NVivo computer software program to organize and sort data.

**Table 1:** Interview participants’ characteristics

Participant No.	Job Title/Role	Visible minority	Sex	Work status - F/T or P/T
1	Trainee	Y	F	F/T
2	Allied Health	Y	F	F/T
3	Allied Health	Y	F	F/T
4	Nurse	Y	F	F/T
5	Manager	N	M	F/T
6	Manager	Y	F	F/T
7	Nurse	Y	F	P/T
8	Support Staff	N	F	F/T
9	Ancillary	N	F	P/T
10	Nurse	N	F	F/T
11	Trainee	Y	F	F/T
12	Ancillary	Y	M	F/T
13	Nurse	Y	F	F/T
14	Allied Health	N	F	P/T
15	Allied Health	Y	F	P/T
16	PSW	Y	F	F/T
17	Nurse	Y	F	F/T
18	Trainee	Y	F	F/T
19	Allied Health	N	F	F/T
20	PSW	N	F	P/T
21	Ancillary	N	F	F/T
22	Support Staff	N	F	F/T
23	Nurse	Y	M	F/T
24	PSW	Y	F	P/T
25	Allied Health	Y	M	F/T
26	Support Staff	N	M	F/T
27	Support Staff	Y	F	F/T
28	Support Staff	Y	F	P/T
29	Manager	N	F	F/T
30	Nurse	Y	F	F/T
31	PSW	Y	F	F/T
32	Manager	Y	F	F/T
33	PSW	Y	F	F/T
34	PSW	Y	F	F/T
35	Allied Health	Y	F	F/T
36	PSW	Y	F	F/T
37	Support Staff	N	F	F/T
38	Nurse	Y	F	F/T
39	Ancillary	Y	M	P/T
40	Ancillary	Y	F	P/T
41	Nurse	Y	F	P/T
42	Ancillary	Y	M	F/T

## Results

The findings indicate that typically, LTC workers maintained health and wellbeing through self-care measures such as healthy eating and exercise, including walking, yoga, swimming, and going to the gym. Thirty-five out of 42 participants (83.3%) reported self-care measures that included exercise, healthy eating, and so forth. These measures were often conducted in group settings or tied to emotional health, suggesting important social and mental health contexts to these behaviors. Some participants also reported that they had the time and resources to grow their own food, and used consumption as a proxy for relaxation and emotional health. However, there were other workers who said that finances were barriers to practicing healthy behaviors.

### Self-care – healthy eating

A few key responses summarize the extent to which healthy eating was practiced, along with the rationale for practicing such self-care, such as coping with work because of how it spilled over into domestic life. For example, a female ancillary worker reported that she had hypertension and pre-diabetes. While this worker attributed her medical conditions to family history, more interestingly, her physician suggested they were likely from her “job”, suggesting that employment and working conditions were responsible for this metabolic syndrome component. Accordingly, in order to cope with this type of work-related stress, she reported that she stopped taking her “work home”. She also practiced healthy eating by making her own food from scratch:

A: “High blood pressure and I think that was my doctor said it’s probably from the nerve from your job. That’s why I decided to separate my – no longer take my work home with me because I think that’s how I must have gotten it. But yeah, I have a high blood pressure. And I’m borderline diabetic but that runs in the family. Hopefully, I won’t get the disease but I probably will because most of the people in my family have gotten it.”

I: “Do you try to eat healthy?”

A: “Oh yeah, I grow my own. I make everything from scratch. I don’t buy any processed food at all because processed food has sugar and it has salt in it. They’re both real bad for you. They have apple ingredients and half of those, they just have funny names for them. But it’s still the same thing. It’s salt and sugar. They just have fancy names for them that’s all. Just call a spade a spade. You don’t have to have 60 trillion names for it. So there’s people who don’t understand what the hell you’re talking about unless you happen to be a professor or a scientist that know all about the stuff. And I’m skinny.” (Participant 9, Ancillary Worker, Female, Non-VM, P/T).

The above participant went on to state that she would go home after work and have a cup of tea, possibly alluding to a relaxation strategy. As another health-conscious practice, she also grew her own food:

A: “I usually just go home and just have a cup of tea and just sit down and think about nice things. The work that I have to do at home. I like to cook and I like to garden […] every day I do my cooking. [name of family member] says, ‘Where are you going?’ ‘I’m going to go enjoy myself.’ ‘Oh, you’re going to cook.’

I: “Oh, what about in the winter? If you’re not able to garden, what do you do if you’re not able to?”

A: “Oh, I grow all my fruits – all my vegetables all year long. […] Like I have my green peppers, my green pepper tree I brought in from outside. It’s still growing. […] I enjoy it.” (Participant 9, Ancillary Worker, Female, Non-VM, P/T).

One quote demonstrates how some workers practiced self-care because they were conditioned to it, and they considered it to be important. For example a Trainee indicated that she engaged in regular physical activity, adequate sleep, and healthy eating habits because she considered them to be really important and; therefore, prioritized these things to the point where she shifted her life around them:

“I’ve always been conditioned-- I guess to like – for exercise and stuff like that. Eating well, sleeping well. That to me is really important. So I make time for that. I sort of shifted my life with it though because now I go to bed super early and I wake up earlier so I get some school work done before I start here because I find it’s just easier for me to do rather than come home to do it because I’m tired.” (Participant 11, Trainee, Female, VM, F/T).

Another participant stated that she tried to eat healthy, but she admitted that she also ate junk food sometimes:

“I am trying [to eat healthy] technically. I'm trying, because I can see, even me, I've never had any problem until 45, I ate anything I wanted, I did anything. But now, I need to watch [it…], but sometimes we're just eating some junk as well.” (Participant 37, Support Staff Worker, Female, Non-VM, F/T)

Although the above participants indicated they ate somewhat health-consciously, a few indicated that they had income and budgeting challenges:

“So it has extended over to where we live, it’s definitely the cost of living is astronomical, you’re going from – I had [dependents] also live with us, so we’re a family of four and groceries can be expensive, especially if you want to eat healthy, right” (Participant 10, Nurse, Female, Non-VM, F/T)

One participant’s response succinctly summarizes how she was sometimes unable to eat healthy exclusively due to costs:

“I've tried to like eat like salad and like going to like the store to buy salad in a box. It's like so expensive. So sometimes, it's like, oh, I don't have the money for that right now. So yeah, it's expensive.” (Participant 15, Allied Health, Female, VM, P/T).

One PSW attributed the inadequacy of the pay to the stagnation of wages and described the effect of this inadequacy upon the quality and variability in the food she was able to serve to her family. In particular, she gave an example of how junk food, such as a bag of fries, is easier and cheaper to purchase than healthy food, such as salad:

A: “[T]he cost of living has gone up, and we haven’t gotten a raise in so many years. I don’t even know. I just -- I don’t even keep track. And it also makes a lot of people mad, because I started six years ago. They’ve worked here 20 years ago, and the pay is the same.” (Participant 20, PSW, Female, Non-VM, P/T).

The same participant went on to state:

A: “The other day, my kids were like, “Oh, mom. Chicken again?” “Well, chicken’s on sale right now. So yeah, that’s what you’re having.“ And I just try and differ it up a little bit. But yeah, for sure. It’s easier to buy a bag of fries than it is a whole salad. Salad’s expensive, but you do what you’ve got to do.” (Participant 20, PSW, Female, Non-VM, P/T).

### Self-care – physical activity

Although a few participants did not participate in physical activity and attributed their thin physique to their metabolism, many routinely reported engaging in physical activity as a form of self-care. This was done in groups or individually. For instance, one worker stated in the interview that she engaged in regular exercise as a social activity with her friends:

A: “So I work out with my friends like every three days.”

I: “In the gym?”

A: “Yeah, in the gym. We do like different [activities on different] days. So like Mondays, we would work on our stomach or something, and then Wednesday, our legs, and then -- and then, like, Friday, we just jog.” (Participant 15, Allied Health, Female, VM, P/T).

Another worker indicated that she engaged in regular, vigorous exercise to clear her mind, and this was done in group sessions, such as in a class:

A: “I’m just going. I like classes. You don’t need to think and you don’t have time to think because it’s the music loud. Everyone jumping and someone, you don’t even… That is why I love classes because you are in, that is, what, 45 minutes, one hour, you are not thinking about anything. If there’s no classes, then treadmills is the other one. Whenever you’re running or you, or just sometimes I’m just listening a book. On my phone, I have books and then I listen and then I’m not thinking about something else [sic].” (Participant 37, Support Staff Worker, Female, Non-VM, F/T).

Another worker said she used walking, listening to music, and swimming as coping strategies to relax and destress herself, despite environmental barriers such as extremely cold weather conditions (which were the conditions in which the interview took place with this worker):

A: “I’m really into music. […] I love music. I’m always listening to music -- when I walk to work, walk home. I swim, too. […] It’s really relaxing.” […] And I go for really long walks. Sometimes I’ll go for a walk for 3 h if I need to just clear my mind. […] I just find walking and listening to music is just a good way to clear your mind.”

I: “Okay. Even in the weather like --?”

A: “Yeah. Yeah. […] I don’t mind. As long as I’m warm, I’m good.” (Participant 14, Allied Health, Female, Non-VM, P/T).

A Personal Support Worker (PSW) reported using exercise after waking up in the morning and before coming in to work:

I: “And you’re on your feet all day. Are you able to -- you’re -- I’m guessing you’re very tired. Are you able to do any […] walking moderately or physical activity?”

A: “Well, what I do in the morning, I get up and I do exercise. I exercise for 45 minutes. […] I do cardio. I have my exercise tapes, so -- and I do it three to four times a week. […] When I go on my break, I try to walk the stairs. But that’s -- sometimes the feet tired, so I forget that.” (Participant 16, PSW, Female, VM, F/T).

One worker described regular exercise as a recreational activity which was pursued during the weekdays, and called it a form of entertainment:

A: “I mean, Monday to Friday I go to the gym. So, like, that’s […] That’s entertainment to me, I guess. That makes it seem like I don’t do anything during the week but it’s, like, my own choosing, right? […] Yeah, I weight train, Monday to Friday.” (Participant 26, Support Staff Worker, Male, Non-VM, F/T).

## Discussion

The data suggests that the LTC workers in the urban region of study typically reported consumption of healthy diet and participation in physical activity in order to influence their health and wellbeing. This study also supports evidence that physical activity participation also occurred in group or social settings, suggesting they are important factors for behavior modification.

The findings reveal that the majority of front line health care workers, many of whom were racialized persons, immigrants, and/or women, relied on particular mechanisms for self-care such as healthy eating and exercise, including walking, yoga, swimming, and going to the gym. While the data suggests that participants routinely engaged in these activities, a few participants reported they did not do so, which was explained by cost-related barriers.

The evidence from this study demonstrates several important points. Firstly, the findings shed light on under-researched areas of how workers engage with health-conscious behaviors in order to access their preferred health and wellness practices and maintain emotional health. This study also provides interesting perspectives as to what care workers perceived as healthy or unhealthy. For instance, processed foods, sugar, and salt were considered unhealthy by some participants while salad was considered healthy by others.

Thirdly, the findings demonstrate that many of the workers must rely upon their own resources to achieve their optimal health and wellbeing, including costs. This is an important point because behavioral interventions are modulated by social and material circumstances which could otherwise impact morbidity and mortality [13]. Occupational health and safety issues among vulnerable groups, such as racialized and immigrant workers in Canada for example, are often associated with particular working conditions, work exposures, or ergonomics issues [45]; however, there might be additional factors that influence patterns of sickness in workers, such as income and social status [13]. Indeed, the work of Magnavita [23] indicates that low wages can drastically reduce the motivation of employees to participate in workplace health promotion. Magnavita [23] further advises that at the level of the organization, time constraints, financial and human resource shortages, lack of flexibility in work organization such as job rotation, as well as attitudes of employees and managers (e.g. reluctance to change work habits and practices) were obstacles in carrying out workplace health promotion for older workers. Thus, it is imperative that workplace health promotion interventions should be addressed from both collective and individual points of view that improve working conditions, the occupational environment, work organization, family, community, and social contexts [23].

Canadian research suggests that (im)migrants, racialized populations, and women are vulnerable to poverty, illness, and diseases related to low income, psycho-social/ chronic stress, and socioeconomic status disparities ([8, 14, 15, 28, 29, 32, 33, 41, 47, 48]). The literature also shows that racialized and immigrant workers are vulnerable to both acute and chronic health problems because of structural issues in the labour market [11, 12, 48] that lead to major health risks such as work-related accidents or illness, mental stress, as well as income inequalities and health inequities [4, 8, 15, 16, 42, 45, 48, 49, 52]. Accordingly, it is imperative that these groups are targeted in order to reduce health inequities.

### How might this research impact on policy or clinical practice in the foreseeable future?

Given the above literature, the findings from this study contribute new information to interdisciplinary occupational health scholarship and contextualize health-conscious behavioral practices from one of the most highly intensive work environments and sectors of employment. The findings are important because the knowledge of personal health practices among workers could reflect resistance and resilience strategies, demonstrate how agency is expressed, as well as illuminating any barriers or limitations. It is increasingly recognized that work influences health and disease in a number of ways, including job-related factors such as income and wages, hours of work, work-load and stress levels, interactions with coworkers, access to paid or unpaid sick leave, and work environments, among other things, all of which impact not only the health and well-being of workers but also their families and communities [19, 47]. Consequently, policies may be introduced to minimize barriers and improve access to these interventions. Magnavita [23] suggests that there are also structural barriers that would need to be addressed. For example, workplace health promotion for older workers is less common in small companies than larger ones due to financial and human resource shortages (ibid).

While this study contributes new knowledge, more work can be done. For instance, there are a number of opportunities for further research, such as examining incomes, income distribution, and as well as factors that impact emotional health, such as reporting of workplace violence, or sleep hygiene. The latter two are important given the nature of Canadian LTC work, which is often carried out in shifts, and where exposures to workplace violence have also been reported. Research further suggests that work-related stress and workloads in the LTC sector can be overwhelming [47]; however, strategies to address these issues are often limited, and would require a holistic approach which considers income, employment, education, i.e. socioeconomic status, and other social determinants of health (SDoH). For example, diet and physical activity are just a few interventions that can modulate worker health and wellbeing. There are also more integrated interventions which seek to collectively address worker safety, health, and well-being, known as total worker health (TWH) initiatives [19]. TWH involve work-related environmental, organizational, and psychosocial factors [6], and include the control of physical, biological, and psychosocial hazards and exposures; organization of work; compensation and benefits; built environment supports; and work-life integration [19]. The TWH initiatives have been advocated through the National Institute for Occupational Safety and Health (NIOSH), the Centers for Disease Control and Prevention (CDC), and various researchers, including those at the Harvard School of Public Health and elsewhere [26, 27, 35, 36, 44]. TWH explores opportunities to protect workers and advance their health and well-being, and that of their families by improving working conditions through workplace programs, practices, and policies [19]. The rationale for the above measures is to reduce the burden on the workforce, and control health care costs and economic costs to society [31].

In order to improve management of care work, such as in the case of the LTC sector selected for this study, there needs to be commitment to total worker health and wellbeing, which involves the home, family, and community of the workers. While individual, behavioral factors were addressed in this study, it would be beyond the scope and scale of this study to investigate structural factors such as work organization, family, community, and social contexts; although these factors would be good starting points of further investigation for future studies.

Furthermore, given the diversity of care workers in the region of study, such approaches would need to be culturally appropriate, and adequate supports must be provided to the workers. This means that not only do services and provisions need to exist, but they also need to be available, affordable, and accessible to the workers who require them. When such services and support systems are made available to workers, they can perform the work better, safely, with less of a personal toll on their health and wellbeing, and with better outcomes for the recipients of care. Additional approaches that would be beneficial if they were to be applied to this and other sectors of employment include: allocating limited resources for provisions of good, stable jobs; decent income; poverty-reduction strategies; and advocating the SDoH ([38, 39]; [7, 34];).

## Conclusions

Behaviour modification such as diet and physical activity are embedded in social, political, and economic realities. More research needs to be done to explore health behaviors in highly stressful occupations, while also advocating for holistic approaches that also improve social and material circumstances for workers. While biomedical interventions often reinforce medicalized, positivist solutions, such as diet, physical activity, and screening/surveillance of vulnerable workers, these interventions are limited unless they include integrated approaches. Workplace health promotion interventions should continue to implement occupational risk prevention; however, it is better if the interventions are participatory and inclusive of workers through a bottom-up approach as opposed to exclusively top-down approaches [23]. Accordingly, an alternative framework that considers emotional health, TWH, and SDoH is needed because there are various structural, organizational, community, social, cultural, and policy factors that play a role in the development of illness and health in workers. Indeed, as Sorensen and Barbeau [43] recognize, commitment to worker safety and health throughout all levels of an organization is critical, and organizational leaders should acknowledge, prioritize, and communicate widely the worker safety and health on the same level as quality of services and products that are delivered by that organization.

## Data Availability

The datasets generated and/or analyzed during the current study are not publicly available due to copyrights, large and multiple file sizes, and because they are being used for further analysis for separate, distinct studies, but are available from the corresponding author on reasonable request.

## Abbreviations

CDC: Centers for Disease Control and Prevention
ERI: Effort Rendered Imbalance
F/T: Full Time
LTC: Long Term Care
NIOSH: Occupational Safety and Health
ORE: Office of Research Ethics
P/T: Part Time
PIRI: Psychological Injury Risk Indicator
PSW: Personal Support Worker
SDoH: Social Determinants of Health
TWH: Total Worker Health
VM: Visible Minority
WHO: World Health Organization
